# The Role of National Immunization Technical Advisory Groups (NITAGs) in the Introduction of Inactivated Polio Vaccine: Experience of the Indonesia and Uganda NITAGs

**DOI:** 10.1093/infdis/jiw601

**Published:** 2017-07-01

**Authors:** Antoinette Ba-Nguz, Alex Adjagba, Toto Wisnu Hendrarto, Nelson K. Sewankambo, Celia Nalwadda, Annette Kisakye

**Affiliations:** 1 Health Policy and Institutional Center (Agence de Médecine Préventive), Paris, France;; 2 Indonesia NITAG;; 3 Uganda National Academy of Sciences;; 4 Uganda NITAG,; 5 Makerere University College of Health Science Uganda; and; 6 WHO Uganda Country Office

**Keywords:** Immunisation Advisory Groups, Indonesia, Uganda, SIVAC Initiative, Inactivated Polio Vaccine.

## Abstract

**Background.:**

National Immunization Technical Advisory Groups (NITAGs) are established by national authorities to provide them with independent, bias-free, objective, and evidence-based advice on vaccines and immunization challenges. As of December 2015, 125 countries have reported having set up an NITAG. The Health Policy and Institutional Development Center at the Agence de Médecine Préventive, a World Health Organization (WHO) Collaborative Center for evidence-informed immunization, through its Supporting Independent Immunization and Vaccine Advisory Committees (SIVAC) Initiative project, provides assistance to low- and middle-income countries in the establishment and strengthening of their NITAGs. The Indonesian NITAG (ITAGI) was formed in December 2006 and Uganda’s (UNITAG) was formed in November 2014. Both Uganda and Indonesia have introduced inactivated polio vaccine (IPV) as part of the Global Polio Eradication and Endgame Strategic Plan (the Endgame plan). The authors reflect on the process and the role played by NITAGs in the introduction of IPV in the routine immunization program and the lessons learned.

**Methods.:**

This commentary is a reflection of the authors’ experience on NITAG’s role as observed in 2 particular local settings and applied to a global public health issue, the polio eradication Endgame plan. The reflection is backed up by the relevant (policy and technical) documents on polio eradication, along with minutes and reports from countries’ ministries of health, immunization programs, WHO, and NITAGs.

**Results.:**

NITAGs are valuable tools for ministries of health to ensure sustainable, evidence-informed immunization policies that are trusted and accepted by their communities. Early engagement with NITAGs also ensures that the adoption of strategies addressing global public health threats at the country level reinforces the national immunization programs. On the other end, when NITAGs are proactive and forward-thinking, they can contribute to a smooth and effective introduction of the above-mentioned strategies. Time and resources are key factors to ensure optimal performance of NITAGs.

Several articles have been published that demonstrate that vaccination is a cost-effective public health intervention. A recent study shows that across 94 low- and middle-income countries, the net benefits of averted treatment costs and lost productivity across the life span of immunized cohorts were worth 16 times the required investment compared to unimmunized cohorts [1]. The World Health Organization (WHO) initiated the Expanded Programme on Immunization (EPI) in 1974 with the initial goal to vaccinate all children against 6 diseases: diphtheria, whooping cough, tetanus, measles, poliomyelitis, and tuberculosis diseases. In 2014, an estimated 86% of children under 1 year of age globally had received at least 3 doses of a diphtheria-, tetanus-, and pertussis-containing vaccine (DTP3) [2]. Additional vaccines (hepatitis B and *Haemophilus influenzae* type b) have been since added to the original 6 antigens recommended. Furthermore, thanks to increased commitment of countries and partners, newer vaccines (rotavirus vaccines, pneumococcal vaccines, human papillomavirus vaccine) are now accessible to children in low-income countries soon after they are available on the market. To take full advantage of the potential of vaccination, the decision-making process at the country level should be evidence-informed and guided by the local context. In view of ensuring sustainability, acceptability, and ownership of the immunization programs, several recommendations [3] have been made to countries to establish independent group of national experts, National Immunization Technical Advisory Groups (NITAGs), to provide unbiased advice to their governments. The Global Vaccine Action Plan–Decade of Vaccines (2011–2020), a global strategy aiming at preventing millions of death through vaccination, considers the existence of an NITAG as a key indicator toward achievement of the strategic objective 1: “All countries commit to immunisation as a priority” [3].

Description of NITAGs’ roles and responsibilities and guidance for their establishment and operations have been published [4]. According to the 2015 WHO–UNICEF Joint Reporting Form, 124 countries reported the existence of an NITAG [5], up from 107 in 2012. Since its inception in 2008, the Supporting Independent Immunization and Vaccine Advisory Committees (SIVAC) Initiative has provided various forms of support to 41 countries in the establishment and strengthening of their NITAGs [6–8].

Lessons learned from SIVAC experience were shared elsewhere [6], and reported that a transparent and credible process of recommendation generation would increase credibility, public trust, and NITAG integration in the decision-making process. While in its role NITAGs will focus on national context, available evidence, and priorities to issue a recommendation, it will also advise on the country contribution to the global public health goal. In this paper, we report on Uganda and Indonesia NITAG involvement in the IPV vaccine introduction to the routine immunization program as part of the global polio Endgame.

## NITAGS’ SPECIFIC ROLE IN IPV INTRODUCTION

### Indonesia

The Indonesian Technical Advisory Group on Immunization (ITAGI) was created under the Republic of Indonesia Ministry of Health Decree, dated 15 December 15 2006, number 1418/ MENKES/ SK/ XII/ 2006. The ITAGI has the following terms of reference:

- Monitor and assess vaccine knowledge improvement, including the development of technology and the production and development of new vaccines;
- Monitor and assess immunization program’s result in order to formulate recommendation for improvement;
- Establish communication and coordination relevant to immunization program with other institutions, professional organizations, and task forces, both at national and regional levels;
- Consult with other experts; and
- Report periodically to the Ministry of Health through the Directorate General for Disease Control and Prevention.

The first Ministry of Health’s request to ITAGI was to provide the Ministry with a recommendation on IPV introduction into the national immunization program. Indeed, country-wide vaccine introduction is a complex issue, as Indonesia is an archipelago consisting of 34 provinces and 497 districts. Immunization services are delivered through 8742 community health centers and 1378 hospitals. A disease-specific task force, Polio Task Force, was created within the ITAGI and assigned to conduct an assessment of the Endgame plan in Indonesia to identify facilitating factors and constraints to take into account for the implementation of the Endgame plan in Indonesia. The results of the assessment were presented at the first ITAGI plenary meeting on 6–7 June 2007 in Jakarta. The ITAGI deliberations concluded that a pilot project on IPV introduction in the routine immunization schedule should be carried out in 1 of the provinces in Indonesia. The pilot project was conducted in Yogyakarta Special Region from 3 September 2007 to December 2012. ITAGI had defined the elements that will inform its recommendation: vaccination coverage, parents’ acceptance, immunological response, and environmental survey. The results of the pilot evaluation were reviewed at an ITAGI meeting in Bogor (West Java Province) with the following data:

- Immunization coverage, ranging from 97–99%;
- No parental refusal for the administration of IPV;
- Immune response detected in 100% of the target population with very good serological titers; and
- Results of the environmental survey that indicated a decreased percentage of polio virus in the environment, from 60% to 1%.

In addition to those scientific findings, ITAGI considered operational issues such as supply availability. ITAGI recommended introducing IPV countrywide, with 3 doses of IPV together with DTP administration, using vaccine available in the public market, while completing the transition to IPVs supplied by Bio Farma.

It is also in NITAGs’ remit to monitor the acceptance and subsequent implementation of their recommendations and consider updating them as needed. Indeed, IPV introduction was discussed at several ITAGI meetings, the last one on 15 May 2015 in Jakarta, with the plan being to potentially introduce 3 doses of IPV. However, WHO’s Strategic Advisory Group of Experts (SAGE) recommended that 1 dose of IPV administered at approximately 14 weeks of age would provide sufficient protection for “risk mitigation” of preventing the reemergence of polioviruses after eradication [9, 10]. Based on new evidence and global recommendations, ITAGI updated the initial schedule from 3 doses of IPV to 1 dose. New international evidence was also discussed during WHO regional and global IPV meetings, where ITAGI members shared their national experiences and learned from other countries. This illustrates the fact that although NITAGs’ role is to provide advice to national governments, they also bring local-level perspectives into the design and implementation of global health strategies.

### Uganda

The Uganda National Immunization Technical Advisory Group (UNITAG) was created by a Ministerial Statement dated 18 December 2014, with the mandate of advising policy makers and program managers to make evidence-based immunization (all ages, all vaccines) policy and program decisions. The UNITAG terms of reference are as follows:

- Conduct policy analyses and determine optimal national immunization policies;
- Guide the national government and the national immunization program on the formulation of strategies for the control of vaccine-preventable diseases through immunization;
- Advise the national authorities on the monitoring of the immunization program so that impact can be measured and quantified;
- Advise the government on the collection of important disease and vaccine uptake data and information;
- Identify the need for further data for policy making; and
- Guide, where appropriate, organizations, institutions, or government agencies in the formulation of policies, plans, and strategies for research and development of vaccine delivery technologies for the future.

UNITAG and ITAGI have similar terms of reference, with the responsibility to provide overall guidance on immunization policies and program performance. In addition, the ITAGI has a role in the country vaccine development.

The UNITAG processes for issuing evidence-based recommendations follow a procedure of question formulation, evidence gathering and quality assessment, and analysis by members to develop consensus recommendations. UNITAG has issued recommendations on the drafting of the Immunisation Bill (which was enacted into law in December 2015), as well as recommendations on proposed introduction of rotavirus vaccine into the routine immunization program, and campaign immunization with meningitis A vaccine for populations in susceptible areas of the country. More recently, the Ministry of Health requested UNITAG to provide advice on prioritization regarding the introduction of new vaccines in the country during the next 5 years and, as such, UNITAG is developing the processes and criteria to be followed in the prioritization decision-making process.

In November 2013, SAGE recommended that all polio-endemic and high-risk countries should establish a plan for IPV introduction by mid-2014 and all oral polio vaccine (OPV)–only using countries have such plan by the end of 2014. An official letter was sent to all health ministries of the countries in December 2013, highlighting the SAGE recommendation, the Global Alliance for Vaccines and Immunization support (where applicable), availability of Technical Assistance, and need to meet Endgame timelines. The letter urged high-level advocacy to make IPV introduction a priority at the political level, identify a WHO focal point at the country level to ensure a decision on IPV introduction by national advisory groups is taken promptly, and set a date for IPV introduction (with a recommendation that an IPV introduction plan be developed by mid-to-end 2014, including vaccine registration and cold chain system).

Following the global decision, Uganda was placed with other tier 2 countries, and was required to develop an IPV vaccine introduction by 1 May 2014. This information was received at the country level during the first quarter of 2014. The UNITAG was not yet created at that time, but due to the commitment of the Ugandan government, the EPI technical committee reviewed several reference documents and immediately started the process of developing the IPV introduction proposal. It was presented to several Ministry of Health (MoH) institutional framework structures that discussed and approved the introduction of IPV into the routine immunization program. Prior to its approval, the proposal to introduce IPV was exhaustively discussed by EPI stakeholders on many occasions during the monthly technical meetings organized by the Uganda National Expanded Program on Immunization (UNEPI). The above decision was reached by the country’s MoH before the UNITAG was formed in November 2014. 

After the creation of the UNITAG, Uganda did not consider it necessary to revisit the IPV introduction decision, and hence did not seek the advice of the UNITAG. Moreover, time to the planned IPV introduction dates was also considered too short and inadequate for the UNITAG (which was, in its early infancy, not able to carry out a thorough evidence-informed due diligence to advise government). However, the MoH was contacted and informed of the policy decision and plans to replace trivalent OPV (tOPV) nationwide by concurrently introducing bivalent OPV (bOPV) and IPV. The UNITAG chair was invited by the MoH to participate in the preparation for and oversight of monitoring and supervision of the withdrawal process of OPV type 2 in the country to ensure compliance to the set global standards. The chairperson served as a member of the National Switch Validation Committee for the polio Endgame activities in the country. Uganda did not consider it necessary to subsequently seek the advice of the newly created NITAG. Likely, Uganda considered that the guidance from WHO was well thought out and of high quality, and IPV introduction being part of a global strategy, the guidance from WHO would be sufficient. Even though the decision to introduce IPV in the country did not benefit from UNITAG advice, the group is now firmly established as a key role player in advising the MoH.

- In summary: Although NITAGs play a role at decision-making process level and less at the implementation phase level, in the case of IPV the roles of the Ugandan and Indonesian NITAGs were essentially to advise on the practical aspects of the introduction of IPV and not on the decision whether to introduce it or not. In the context of the polio Endgame plan, policy advice from the NITAGs on the use of IPV may not have been relevant. The fact that the NITAGs of the 2 countries were nevertheless involved in the process of preparing the IPV introduction reflects the fact that these NITAGs are integrated into their institutional systems—an indicator of their sustainability.

### NITAGs Added Value to the IPV Introduction Process

National ownership in the vaccine introduction decision is paramount to the credibility and sustainability of the immunization programs. Even in global health problems, a clear understanding of the issues by countries and the domestication of global recommendations will ensure involvement with a sense of shared responsibility.

NITAGs’ role is also to help countries fulfill their responsibilities with regard to global issues. The SIVAC Initiative, as per its mandate, supports NITAGs for optimal functioning. One key SIVAC strategy is to facilitate networking and experience sharing among NITAGs, so that countries can learn from each other and take concerted action on common issues. SIVAC facilitated a joint meeting of the Benin, Côte d’Ivoire, and Senegal NITAGs on 27 February 2014 in Cotonou. Among other objectives (ie, discussing their priorities for 2014, their experiences, and their lessons learned during the first months of activity), the 3 West African NITAGs were to discuss their recommendation on IPV introduction into routine immunization. The meeting helped the 3 recently established NITAGs appreciate their advisory role to their governments in the implementation of global health strategies [11]. Experience sharing will be highly facilitated if all NITAGs collaborate through both regional networks and the global NITAG Network established in May 2016 [12].

The ITAGI’s process for issuing a recommendation on IPV introduction in routine immunization allowed for the generation of local evidence (scientific and programmatic) that formed the basis of the recommendation to introduce IPV in routine immunization. This approach facilitated the IPV introduction, as the recommendation also considered social, economic, and cultural aspects of the intervention. This example illustrates the needs to have a strong secretariat that coordinates all the preparatory work, as well as having strong technical experts in the NITAG. The need to consider socioeconomic aspects also aligned with the SIVAC Initiative strategy to reinforce the technical capacity of NITAG members, through local training workshops and technical support. This case also illustrates the fact that a rigorous scientific recommendation development process is a key factor to aid NITAG integration by the national authorities.

In Uganda, although the decision for introducing IPV was made before the UNITAG was established, the MoH invited a member of the Ugandan NITAG to participate in the preparation for and oversight of monitoring and supervision of the withdrawal process of OPV type 2 in the country, to ensure compliance to the set global standards. This speaks to the acknowledgment by authorities that the principles of independence and rigor to which UNITAG members abide in their operations are of great value for the credibility of the immunization program. UNITAG’s participation added value by overseeing the switch validation processes undertaken by UNEPI to ensure transparency and compliance to the agreed procedures. In addition, UNITAG had a greater appreciation of the state of the immunization program (including the cold chain) through visits made to individual vaccination centers. In the future, UNITAG can play similar roles in monitoring the implementation and impact of policy decisions such as the immunization bill referred to above.

### Lessons Learned

The 2 experiences reported here, albeit in different contexts, point to the fact that national authorities call on their NITAG for advice, even regarding worldwide issues for which the strategy is coordinated at the global level.

For ITAGI, the early involvement by the national authorities allowed for implementation of their processes in order to issue a timely, evidence-based recommendation: a specific task force was formed to assess the problem and identify data needed to support a recommendation. The absence of critical local data led to a recommendation to conduct a pilot study, which then provided the evidence for sound and comprehensive advice. Early involvement of the ITAGI also allowed for several meetings to deliberate and review the evidence before the recommendation was submitted to the MoH of the Republic of Indonesia through the Directorate General of Communicable Disease Control.

The decision to introduce IPV in Uganda did not benefit from the country’s NITAG expertise and local contextual considerations, as the NITAG was not established at the time when the plan to introduce IPV was made. Countries that have an NITAG need to begin to think out of the box (despite the global targets) and sufficiently develop the NITAG to have the capacity and authority to carry out tasks that are expected of them, so that national governments can make their independent evidence-informed decisions, even when having to meet a WHO or other global guideline. Adequate time and supporting resources must be provided to ensure optimal performance of the NITAG.

## Notes

***Acknowledgments.*** We thank the WHO colleagues from WHO headquarters, all colleagues from the regional offices, and the immunization partners involved in NITAG strengthening at the country level.

***Disclaimer.*** The SIVAC Initiative resides within a separate legal entity within AMP that receives no support from vaccine manufacturers. The SIVAC Initiative is funded entirely by a grant from the Bill & Melinda Gates Foundation and GAVI, the Vaccine Alliance.

***Supplement sponsorship***. This work is part of a supplement coordinated by the Task Force for Global Health with funding provided by The Bill and Melinda Gates Foundation and the Centers for Disease Control and Prevention.

***Potential conflicts of interest.*** All authors: No reported conflicts. All authors have submitted the ICMJE Form for Disclosure of Potential Conflicts of Interest. Conflicts that the editors consider relevant to the content of the manuscript have been disclosed.
